# Disruption of brain connectivity in acute stroke patients with early impairment in consciousness

**DOI:** 10.3389/fpsyg.2013.00956

**Published:** 2014-01-02

**Authors:** Yuan-Hsiung Tsai, Rui Yuan, Yen-Chu Huang, Mei-Yu Yeh, Ching-Po Lin, Bharat B. Biswal

**Affiliations:** ^1^Department of Biomedical Imaging and Radiological Sciences, National Yang-Ming UniversityTaipei, Taiwan; ^2^Department of Diagnostic Radiology, Chang Gung Memorial Hospital at Chiayi, College of Medicine and School of Medical Technology, Chang-Gung UniversityTaoyuan, Taiwan; ^3^Department of Biomedical Engineering, New Jersey Institute of TechnologyNewark, NJ, USA; ^4^Department of Neurology, Chang Gung Memorial Hospital at Chiayi, College of Medicine and School of Medical Technology, Chang-Gung UniversityTaoyuan, Taiwan; ^5^Institute of Neuroscience, National Yang-Ming UniversityTaipei, Taiwan

**Keywords:** fMRI, resting state, stroke, brain connectivity, resting state functional connectivity

## Abstract

Impairment in consciousness is common in acute stroke patients and is correlated with the clinical outcome after stroke. The underlying mechanism is not completely understood, with little known about brain activity and connectivity changes in acute stroke patients having impaired consciousness. In this study, we investigated changes in regional brain activity and brain networks of consciousness impaired stroke patients, as well as the amplitude of spontaneous low frequency fluctuation (ALFF) of each time series. Regional homogeneity (ReHo) of each voxel was measured, and resting state network analysis was consequently conducted. Results from this study demonstrate that, compared to normal subjects, the intensities of ALFF and ReHo, as well as the strength of the default mode network (DMN) connectivity, were significantly decreased in the precuneus and posterior cingulate cortex regions among stroke patients with impaired consciousness. Furthermore, the strength of the DMN was highly correlated with differences in the Glasgow Coma Scale (GCS) scores between the onset time and the scanning time. Results from this study suggest that the resting state fMRI is a feasible tool for the evaluation of acute stroke patients with an early impairment of consciousness. The detailed mechanisms, implications of these brain activities and networks exhibiting changes will require further investigation.

## INTRODUCTION

Early impairment of consciousness is common in acute stroke patients. A number of studies have demonstrated the existence of a relationship between the level of consciousness impairment and the mortality/morbidity rate after stroke (Frankel et al., 2000; Weir et al., 2003; Lansberg et al., 2011). An infarction or hemorrhage in anatomic regions that maintain arousal, such as the brainstem and the thalamus, may cause an early impairment of consciousness (Bogousslavsky et al., 1988; Amici, 2012). Large intracranial hemorrhaging or infarction with edema, and mass effect might also cause the midline shift. The degree of this shift has been shown to be correlated with the level of consciousness (Ropper, 1986). The proposed mechanism for such impairment has been associated with a direct destruction or compression of the reticular system (Steriade, 1996; Cucchiara et al., 2004). Although, some patients regain consciousness; others may have complications such as a prolonged coma or vegetative state. However, previous studies have yet to examine the altered state of consciousness in stroke patients and its relationship to the brain’s activity and functional connectivity.

Recent advances in brain imaging techniques have expanded our understanding of how the brain functions. For example, changes in the blood oxygen level dependent (BOLD) signal during rest using functional magnetic resonance imaging (fMRI) have been shown to be highly associated with the connectivity of functionally related regions of the brain. Resting-state fMRI has been used to demonstrate spatiotemporal correlations within functional networks during rest (Biswal et al., 1995). One of the primary quantitative properties of the resting-state BOLD signal is the amplitude of low frequency fluctuations (ALFF), which measures the total power within the range of 0.01 and 0.1 Hz (Zang et al., 2007). Zang et al. (2007) found that children with ADHD showed reduced ALFF amplitude in specific and relevant regions of the brain. Similarly, Wang et al. (2012) found significant differences in ALFF in brain regions of patients with severe depression. We have previously shown that ALFF correlated significantly with hypercapnic fMRI response in healthy subjects (Kannurpatti et al., 2010; Di et al., 2012). These studies demonstrate the feasibility of using ALFF as a neurophysiological index to show group differences between healthy controls and a number of different clinical populations.

Another quantitative property of resting fMRI is regional homogeneity (ReHo), which characterizes the similarity of local brain activity across a region. In fMRI studies, this measure is obtained by measuring Kendall’s coefficient of concordance (KCC) of a cubic cluster of 27 voxels, with the central voxel of every cubic cluster assigned the derived KCC value. ReHo has also been used in several clinical studies, including attention deficit hyperactivity disorder (ADHD; Wang et al., 2013), Alzheimer’s disease (Zhang et al., 2012), and Parkinson’s disease (Wu et al., 2009).

The default-mode network (DMN; Greicius et al., 2003) is a well-established functional network that is observed during the resting-state imaging. It is generally suppressed during task activation states such as those requiring attention (Uddin et al., 2010) and decision making (Buckner et al., 2008). The DMN is typically comprised of the posterior cingulate cortex (PCC), the precuneus, the inferior parietal, and the medial prefrontal cortex regions. Functional disconnection of the DMN tends to be associated quite strongly with an impairment of consciousness, since the DMN is important in the genesis of awareness (Vanhaudenhuyse et al., 2010; Soddu et al., 2011; Boly et al., 2012; Fernandez-Espejo et al., 2012).

The current study uses resting-state fMRI to achieve two specific goals. The first goal is to characterize the resting state signals by using specific quantitative measures, including ALFF and ReHo, in order to differentiate between stroke patients and health subjects. The second goal is to determine whether the properties of the DMN vary between stroke patients with impaired consciousness and normal healthy subjects. To assess these network properties, spatial independent component analysis (ICA) and dual regression are used to calculate the resting state functional connectivity (RSFC) parameters. We hypothesize that the early alteration of regional brain activity and DMN connectivity play a key role in determining conscious status of acute stroke patients, and that the resting fMRI approach may be used as an imaging marker to estimate the subject’s conscious status and to predict the recovery of consciousness.

## MATERIALS AND METHODS

### SUBJECTS

This study was part of an integrated stroke project at Chang Gung Memorial Hospital, Taiwan, and was approved by the Institutional Review Board of Chang Gung Memorial Hospital. Informed consents were obtained from all the patients’ relatives. Fifteen unconscious patients suffering from their first stroke with no history of any neurological deficits were enrolled. Patients with any contraindications to undergoing the MRI scans and patients requiring emergency surgery were excluded. The Glasgow Coma Scale (GCS) scores and motor response scores were estimated by an experienced emergency physician within 24 h of the stroke. The demographic and clinical characteristics for the included stroke subjects are presented in **Table 1**. In addition, 19 healthy subjects (age range from 77 to 88 years, average 81 + 4.11) without a history of neurological disease or any pathological findings in conventional MRI scans were enrolled.

**Table 1:** Summary of clinical characteristics and voxel number of default mode network of the stroke patients (*n* = 15).

Subject	Age (years)	Gender	Stroke subtype	Lesion size, cm^3^	Lesion side	Lesion location	GCS score at admission	Motor response score at admission	GCS score at discharge	Motor response score at discharge	Duration of hospital stay, day	Voxel number of DMN
1	85	F	Ischemia	101	R	MCA territory	11	5	14	6	4	1849
2	77	M	Ischemia	219	R	MCA territory	10	5	11	6	22	2241
3	86	F	Ischemia	121	R	MCA territory	9	4	9	4	10	1554
4	77	M	Ischemia	285	L	MCA territory	12	5	11	6	10	1580
5	81	F	Ischemia	203	L	MCA territory	13	5	9	4	30	1070
6	89	F	Ischemia	616	R	MCA territory	7	5	6	4	28	1241
7	89	F	Ischemia	9	L	Putamen	12	5	12	5	4	1882
8	79	M	Hemorrhage	47	L	Putamen	10	5	10	6	13	1722
9	59	M	Hemorrhage	54	L	Putamen	9	4	11	6	53	2441
10	46	M	Hemorrhage	62	L	Putamen	3	1	11	6	9	3018
11	62	M	Hemorrhage	68	L	Putamen	10	5	14	6	36	2517
12	80	F	Hemorrhage	70	L	Thalamus	10	5	10	5	39	2657
13	50	M	Hemorrhage	57	L	Thalamus	6	4	7	4	33	1887
14	59	M	Hemorrhage	75	L	Putamen	12	5	14	6	24	2079
15	25	F	Hemorrhage	27	L	Putamen	9	5	14	6	25	2311

### MRI INSTRUMENTATION AND PROCEDURES

All data were collected using a 3 T Siemens Vario MRI system (Siemens Medical System, Erlangen, Germany) with a 32 channel head coil. The MRI scans were all performed within 24 h of the stroke. The imaging procedure for each subject included obtaining T1-weighted anatomical images with a gradient echo sequence (TR = 3500 ms, TE = 2.87 ms, FOV = 220 mm, matrix = 256 × 256 × 160), resulting in a spatial resolution of 0.9 mm × 0.9 mm × 1.0 mm. Additionally, a gradient EPI sequence, sensitive to BOLD contrast, was also obtained (TR = 2500 ms, TE = 27 ms, FOV = 220 mm, matrix = 64 × 64 × 36, slice thickness = 4 mm). Each scan consisted of 240 image volumes and lasted for 10 min and 7 s. Healthy subjects were instructed to stay awake and relaxed with their eyes closed during the resting state scan.

### IMAGE PROCESSING

All fMRI data from each of the subjects was processed using Analysis of Functional NeuroImages (AFNI) software^1^ and the SPM8 package^2^. All data sets were processed using an image registration algorithm for the detection and correction of motion related signal changes. Any subject that exhibited head motion greater than 2 mm was discarded from further analysis. Furthermore, six motion parameters and their Euclidean norm of motion derivatives were regressed out from all time series of each subject. All time series were also linearly detrended to correct for any linear drifts. The anatomical images were partitioned into gray matter, white matter (WM) and cerebrospinal fluid (CSF). Each subject’s deformation field map obtained from the structural image was applied to the functional images for normalization into the Montreal Neurological Institute (MNI) space (resampled at 3 mm^3^ resolution). For each time series, the first five principle components from both WM signals, and CSF signals were regressed out. A band-pass filter ranging from 0.01 to 0.1 Hz was applied to each time series. All data sets were then smoothed using an 8 mm FWHM filter.

### ALFF MAP

Amplitude of spontaneous low frequency fluctuation was calculated using the Resting-State fMRI Data Analysis Toolkit V1.7 (REST)^3^. For each voxel, a discrete Fourier transform was performed on the resting-state time series. The ALFF was computed by measuring the average square root of the total power spectrum between 0.01 and 0.10 Hz on a voxel by voxel basis.

### REHO MAP

Regional homogeneity was calculated in a voxel-wise manner using KCC among the time series of a centered cubic cluster of 27 voxels. A large ReHo value for a given voxel indicates a high local synchronization of resting state fMRI signal among neighboring voxels, and vice versa.

### INDEPENDENT COMPONENT ANALYSIS

Independent component analysis was used to study the resting state network patterns across brain. First, the resting scans of all healthy controls were concatenated along the time axis into a single 4D dataset. This 4D dataset was decomposed by MELODIC into 20 spatio-temporal components, a set of statistically independent sources of the resting state signal represented by large-scale patterns of co-activated voxels. Based on visual comparison with previously identified resting state network patterns (Biswal et al., 2010; Cole et al., 2010), 10 RSN components were selected for further analysis.

Dual regression was used to derive individual resting-state networks for each subject from each group component (Beckmann et al., 2005). Each spatially independent component map produced in the group-level ICA has a corresponding temporal signal. These temporal signals are used in a second regression for each individual in order to identify the spatial map per subject identifiable with that group component. Following standard steps, the regression betas from the individual level RSNs were then transformed to *Z*-scores.

### STATISTICAL ANALYSIS

To determine group differences ALFF, ReHo and functional network connectivity, a two-sample *t*-test was conducted within a general linear model framework. This was used to determine locations of voxels of significant differences between stroke patients and healthy subjects within the DMN, such as within the precuneus and the temporo-parietal junction. Analyses of correlation between clinical measurement and DMN voxel number were performed with bivariate correlations. The correlations were presented with Spearman rank correlation coefficients. A *p*-value of less than 0.05 was deemed as a significant correlation. All statistical analyses were performed using Statistical Product and Service Solutions (SPSS, Version 18).

## RESULTS

### AMPLITUDE OF LOW FREQUENCY FLUCTUATIONS AND REGIONAL HOMOGENEITY ANALYSIS

**FIGURE 1:**
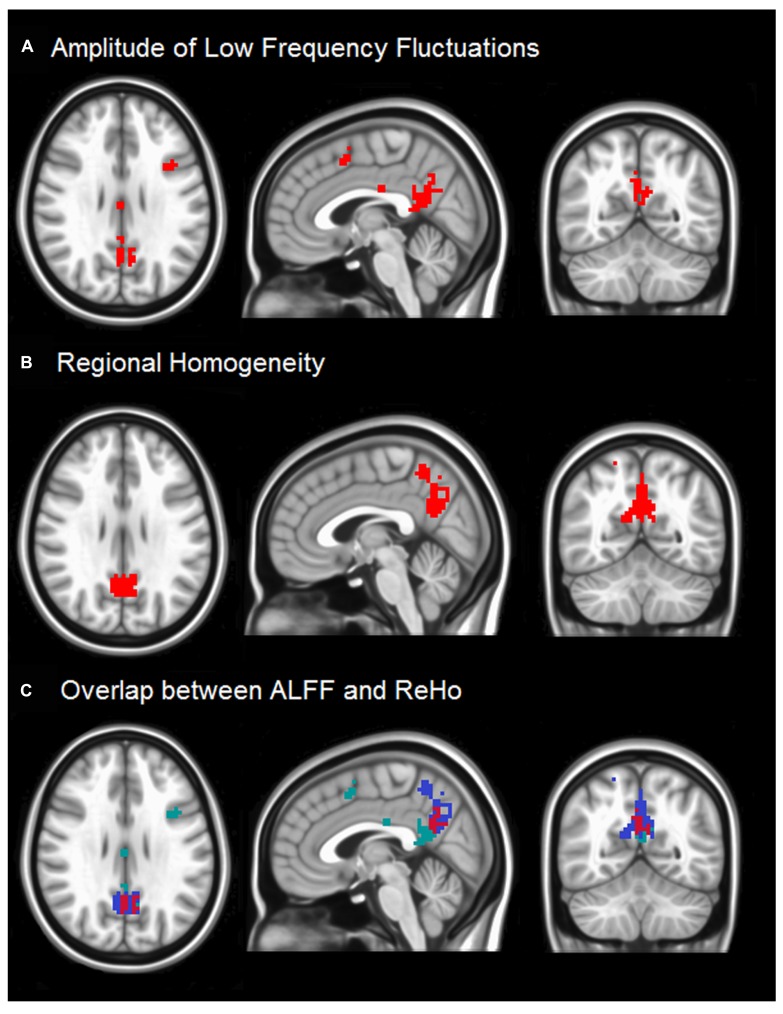
**(A)** The two sample *t*-test result of ALFF between two groups with the threshold at corrected *p* < 0.05 is shown. The regions around PCC (highlighted in red) indicate that stroke patients with impaired consciousness have lower ALFF than matched normal controls. **(B)** Two sample *t*-test results of ReHo between two groups at *p* < 0.05 is shown. The results were threshold at corrected *p* < 0.05. The red regions imply that stroke patients with impaired consciousness have lower regional homogeneity than the normal controls. **(C)** The overlaps between ALFF results and ReHo results are in the red region. The green and blue regions show unique ALFF and unique ReHo, respectively.

**Figure 1** demonstrates differences in ALFF (**Figure 1A**) and ReHo (**Figure 1B**) between healthy subjects and stroke patients with impaired consciousness. Significant group differences appear in the precuneus for ReHo values in the PCC for ALFF. Other brain regions do not reveal significant difference between these two groups. Both the precuneus and PCC regions are considered to be inside the DMN, and highly involved with states of consciousness.

The slight difference in results between ALFF and ReHo may be attributable to previous findings that ALFF maps are more vulnerable to the effects of large vessels than ReHo maps (Di et al., 2012; Yuan et al., 2013). Moreover, several voxels in the medial frontal cortex (MFC) region also show significant differences in ALFF but not in ReHo. The reason may be ALFF measures the power of time series from voxel to voxel, while ReHo measures similarity, which puts all neighboring voxels into consideration. Thus ALFF detects voxel-wise signal differences in the ALFF; ReHo differences represent differences in connectivity among a small group of neighboring voxels.

### RESTING STATE NETWORK ANALYSIS

**FIGURE 2:**
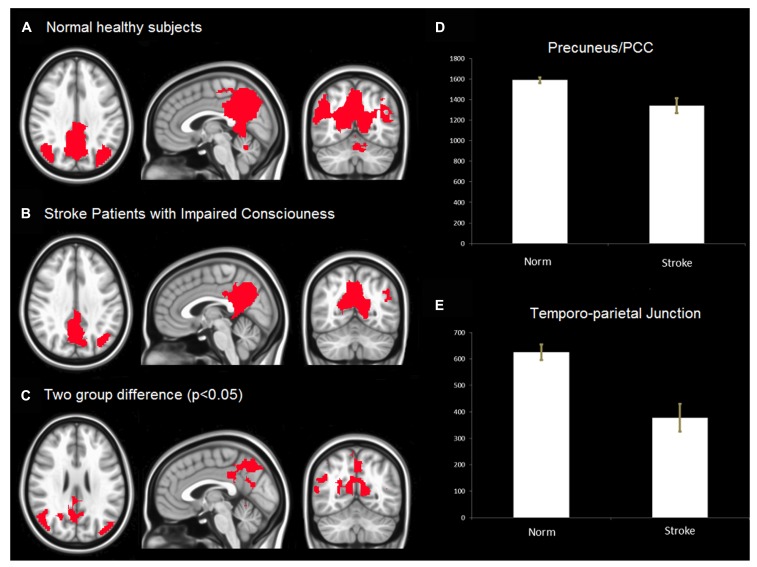
**(A)** Default Mode Network (DMN) in healthy controls. The result was threshold at corrected *p* < 0.05 rendered on MNI152 template. **(B)** DMN of stroke patients with impaired consciousness threshold at corrected *p* < 0.05. The result was overlaid on MNI152 template. **(C)** The group difference of DMN. The result was threshold at corrected *p* < 0.05 and rendered on MNI152 template. **(D,E)** voxels (*T* > 3.2) number of default mode network in PCC/Precuneus, temporo-parietal junction between stroke patient with impaired consciousness and healthy controls. The results show that the number of voxels (*T* > 3.2) of normal health is significantly more than that of stroke patients with impaired consciousness (*p* < 0.05) in both PCC/precuneus and temporo-parietial junction.

In **Figures 2A, B** show the spatial extent of the DMN for the healthy subjects and stroke patients with impaired consciousness. **Figure 2C** illustrates significant differences in the spatial extent of the DMN between the two groups. Among patients with impaired consciousness, a significant reduction in the activity of the PCC, the precuneus and the inferior parietal cortex can be observed (**Figures 2D, E**).

**FIGURE 3:**
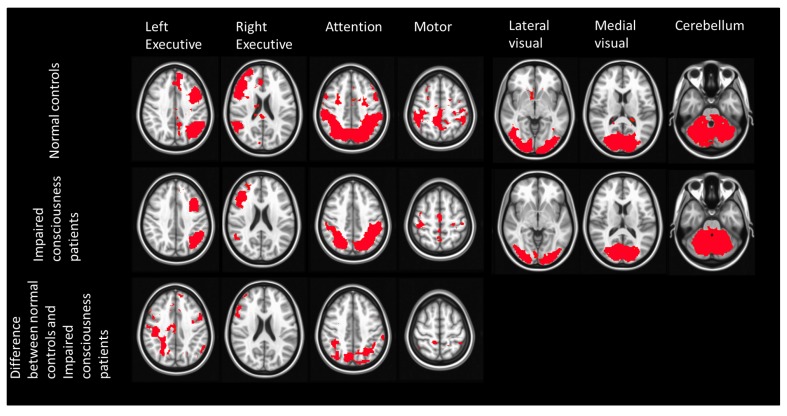
**Spatial maps of 7 RSNs in normal controls and stroke patients with impaired consciousness.** The first two rows show the result of one sample *t*-test of each RSN from both groups (*p* < 0.05). The last row shows the two sample *t*-test result of the left executive network, right executive network, attention network, motor network (*p* < 0.05). No significant difference was found between these two groups in the lateral visual network, medial visual network, and cerebellum.

Considering that the results of changes in DMN connectivity in this study may be mainly due to the effect of stroke instead of conscious impairment, we also investigated the differences of other resting state networks (**Figure 3**). The networks that show significant differences, including the executive network and the attention network, are all related to consciousness and awareness. To further address the role of DMN in consciousness, we calculated the normalized volume of DMN for each subject.

### CORRELATION OF RSFC TO CLINICAL MEASUREMENTS

**FIGURE 4:**
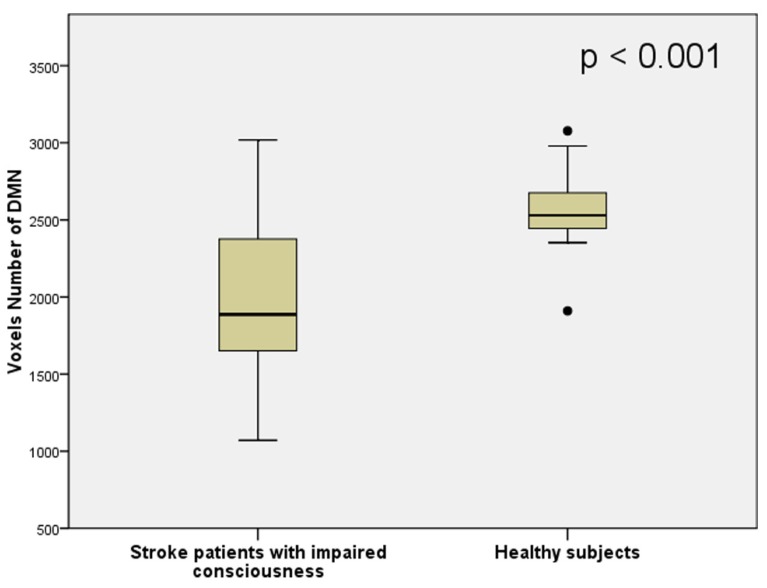
**The number of voxels inside the DMN region of stroke patients with impaired consciousness and healthy controls**.

**FIGURE 5:**
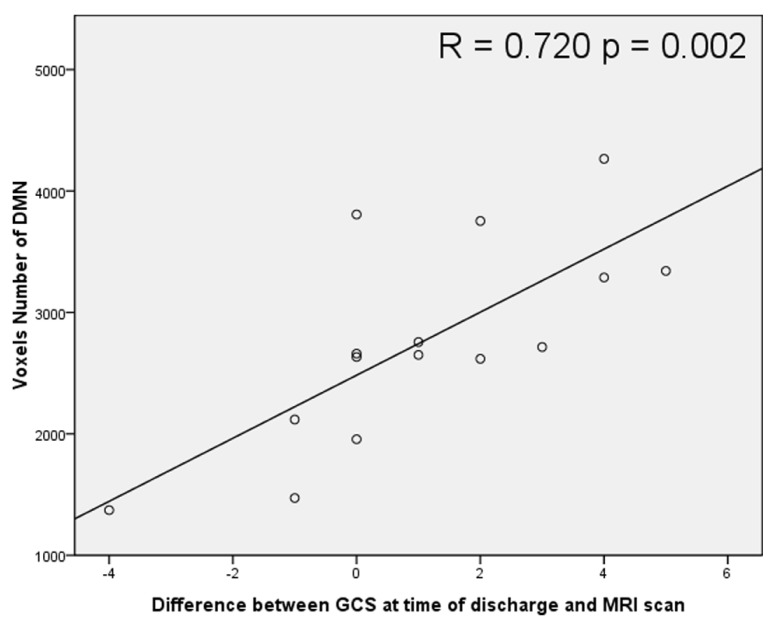
**Scatter plot correlation between voxel number inside the DMN and the difference between Glasgow Coma Scale (GCS) scores at the time of discharge and at the time of MRI scanning**.

The average volume of the DMN (measured by counting interior voxels in MNI space) was significantly lower in stroke patients with impaired consciousness than in healthy subjects (*p* < 0.001; shown in **Figure 4**). There were no significant correlations between DMN voxel number and GCS at the time of the MRI scan (*R* = -0.296; *P* = 0.284) nor at the time when the patient was discharged (*R* = 0.468; *P* = 0.078). A significant correlation between voxel number in the DMN with the difference of GCS at the time of the MRI scan and at the time of the discharge (*R* = 0.720; *P* = 0.002) was observed (shown in **Figure 5**).

## DISCUSSION

Using resting-state fMRI, this study compared the regional brain activity between stroke patients with impaired consciousness and normal healthy subjects by measuring LFFs and the DMN connectivity. To the best of our knowledge, this is the first study using ALFF and RSFC to evaluate brain activity and DMN connectivity of stroke patients with impaired consciousness. Stroke patients in this study had suffered localized brain damage via ischemia or hematoma. Damage from these phenomena is significantly different than that caused by traumatic brain injury or hypoxic brain injury, which are commonly reported in prior studies of conscious disorder. Additionally, vascular pathologies, such as hypertensive angiopathy, amyloid artheriopathy atherosclerosis, or arthritis, are often encountered in stroke patients (Gregoire et al., 2011). These vascular lesions may affect the BOLD signals, thus fMRI studies of stroke patients with impaired consciousness may improve our understanding of consciousness as a whole.

Early impairment of consciousness is associated with poor functional outcome for stroke patients, and acute impairment of consciousness affects whether patients recover or enter a prolonged coma or a vegetative statue. Thus, further understanding of how brain activity and connectivity change after stroke onset and how it affects the patient’s consciousness is important.

### ALTERED AMPLITUDE OF LOW FREQUENCY FLUCTUATIONS IN STROKE PATIENTS WITH IMPAIRMENT OF CONSCIOUSNESS

In contrast to the functional connectivity methods that measure temporal synchronization between brain regions, the ALFF and ReHo approaches performed here localize the brain’s functional alterations. These analyses provide additional information to understand the disturbances of neural networks demonstrated in fMRI studies in patients with impaired consciousness. Using both the ALFF and ReHo measures, we found that among the stroke patients with impaired consciousness, the ALFFs were significantly reduced mainly in the precuneus and adjacent PCC regions.

The precuneus is among the most active cortical regions in the DMN during conscious resting state, and along with the adjacent PCC, has been known to show deactivation in a number of pathophysiological measures of altered states of consciousness. For instance, the precuneus, along with the lateral parietal and prefrontal cortices, was found to be significantly less active during slow-wave and rapid eye movement sleep than during rest (Maquet et al., 1996, 1999). Since the impaired consciousness of self and the environment represents a key feature shared by the different sleep stages, these observations might provide evidence for the role of the precuneus in conscious processes. Anesthetic agents have been found to decrease cerebral blood flow to the precuneus in a dose dependent manner (Kaisti et al., 2002). Another study showed disruption within the functional networks of the PCC-precuneus region and within the non-specific thalamus during general anesthesia (Hudetz, 2012). These studies indicate that anesthetics result in behavioral changes via an effect within specific neuronal networks that regulate arousal and consciousness, including the precuneus. Furthermore, in a study of hypnosis, it was found that relative cerebral blood flow decreased in the precuneus, PCC and right inferior parietal regions (Rainville et al., 1999). In particular, deactivation of the precuneus, was considered to be an important metabolic feature of this altered state of consciousness.

Taken together with the widespread connectivity pattern differences, these findings provide strong, albeit preliminary evidence that the networks to which the precuneus belong may be parts of the neural network serving self-awareness and conscious experience. The precuneus may play a central role in the functional network correlated to consciousness. In this study of stroke patients with impaired consciousness, we have reinforced the known importance of the activity of the precuneus and PCC in the genesis of awareness and maintenance of consciousness.

### DEFAULT MODE NETWORK DISRUPTION IN STROKE SUBJECTS WITH IMPAIRMENT OF CONSCIOUSNESS

Using data-driven ICA, we find disrupted DMN connectivity among stroke patients with impaired consciousness. The connectivity among the PCC, the precuneus, the medial prefrontal, and the inferior parietal regions is decreased among stroke patients. High DMN connectivity has been consistently found in healthy subjects using ICA analysis of resting fMRI data (Meindl et al., 2010). Disruption of the DMN has been linked to pathophysiological conditions with various levels of reduced cognitive function, for instance in cases of epilepsy (Danielson et al., 2011), Alzheimer’s disease (Greicius et al., 2004), depression (Greicius et al., 2007), and schizophrenia (Zhou et al., 2007). Disruption of the DMN was found to be associated with behavior measurement and cognitive performance. Studies of patients with severe traumatic brain injury, including minimally conscious and vegetative state patients, showed disruption of DMN connectivity (Boly et al., 2008; Vanhaudenhuyse et al., 2010; Sharp et al., 2011). The decreased connectivity between the medial prefrontal and PCC/precuneus areas has been observed in vegetative patients, with restored metabolism in the precuneus after conscious recovery (Laureys et al., 1999). Another recent study of patients with different levels of disordered consciousness showed significant impairment in all of the pathways within the DMN (Fernandez-Espejo et al., 2012). These previous studies, combined with the present results, support the conclusion that disruption of the DMN in stroke patients is associated with the impairment of consciousness, rather than with the alteration of cerebrovascular signal.

### DEFAULT MODE NETWORK CONNECTIVITY AND THE SEVERITY OF CONSCIOUS IMPAIRMENT

The GCS is a reproducible and objective neurological scale that has been used to record the conscious state of a person for initial and subsequent assessment (Teasdale and Jennett, 1974). Initially used to assess level of consciousness after head injury, the GCS has become the method of choice after acute neurological insult, for documenting neurologic findings over time and for predicting functional outcome (Fischer and Mathieson, 2001). In the present study, the DMN volume was positively correlated with the change of GCS score over time (value at the time of discharge minus that at the time of the MRI scan).

Default mode network connectivity is correlated with the behavioral signs of awareness in patients with prolong conscious disorders such as minimally conscious state and vegetative state (Fernandez-Espejo et al., 2012; Soddu et al., 2012). To our knowledge, the present is the first study to show the correlation of GCS changes and DMN connectivity in acute neurological illness. It is important to note that the meaning of an impairment of consciousness is different in an acute stage than in a chronic stage (Ohta, 2005). Patients suffering from acute neurological illness such as an infarction, spontaneous hemorrhaging, or traumatic brain injury may experience transient or prolonged impairment of consciousness. The duration and the level of consciousness impairment during the acute stage are important factors for the patient’s prognosis (Weir et al., 2003; McNett, 2007). However, in some patients, the level of consciousness is difficult to estimate. For example, patients who suffer from high cervical cord injury, or other severe limb injury cannot cooperate with the motor response measurement; aphasia or intubation prevents verbal responses from being accurately estimated, and severe eye injury does not allow eye opening/closing methods of evaluation (Barlow, 2012). From the results of this study, the DMN may be considered a promising marker for the prognosis of conscious status.

### DIFFERENCE OF NETWORK CONNECTIVITY BETWEEN STROKE AND HEALTHY SUBJECTS

In a recent study, Park et al. (2011) studied the longitudinal effects of RSFC in stroke patients. To test the hypothesis that the alteration of DMN connectivity in stroke patients found in the present study may not be related to impairment of consciousness but instead to the effects of stroke, we also reanalyzed the other resting state networks with ICA method (**Figure 3**). **Figure 3** demonstrates significant differences of network strength between stroke unconscious patients and healthy subjects. These changes were noted in bilateral executive, attention, and sensorimotor networks. On the contrary, no significant difference was found in medial and lateral visual and cerebellar networks. Thus, the DMN differences between stroke unconscious patients and healthy subjects appear to not be due solely to global vascular effects originating from stroke, as there were several networks in stroke patients had properties similar to those of the healthy subjects.

Moreover, the networks that showed differences in this study, including executive, attention, and DM networks, have also been reported to be related to consciousness and awareness in previous publications (Vanhaudenhuyse et al., 2010, 2011). In addition, the significant correlation of the networks strength with change of GCS between time of discharge and time of MRI scan was only obtained in DMN.

### LIMITATIONS AND FUTURE DIRECTIONS

This study, since it is preliminary in nature, has several limitations. First, as we mentioned in the introduction section, stroke lesions in specific anatomic regions and large lesions with mass effect and midline shift are highly correlated with the level of consciousness. Thus, further investigations enrolling subjects with more specific lesion locations can clarify how the lesion locations disrupt brain network connections and LFFs. Second, a longitudinal study including resting state fMRI scans in a follow-up stage or after patients recover consciousness may be helpful in understanding brain plasticity following impairment of consciousness. In addition, it is necessary to further study whether the change of DMN is due mainly to the impairment of consciousness or to other physiological effects.

## CONCLUSION

This resting state fMRI study’s findings identify specific declines in ALFF and ReHo as well as disruptions of DMN in stroke patients with an early impairment of consciousness. In the context of previously reported decline of the regional blood flow and the disconnections of DMN, resting state fMRI has been shown to be a feasible tool for evaluating stroke patients with a conscious disorder. Further study of larger samples of patients in order to investigate the detailed mechanisms and predictive value of the changes in network connectivity to patient’s outcome is necessary.

## Conflict of Interest Statement

The authors declare that the research was conducted in the absence of any commercial or financial relationships that could be construed as a potential conflict of interest.

## Footnotes

1 http://afni.nimh.nih.gov/afni; version: January 2012

2 http://www.fil.ion.ucl.ac.uk/spm/ (date last accessed 13 January 2012)

3 http://www.restfmri.net (date last accessed 25 May 2012)
